# Smartwatch-Based Interventions for People With Dementia: User-Centered Design Approach

**DOI:** 10.2196/50107

**Published:** 2024-06-07

**Authors:** Doreen Goerss, Stefanie Köhler, Eleonora Rong, Anna Gesine Temp, Ingo Kilimann, Gerald Bieber, Stefan Teipel

**Affiliations:** 1 Department of Psychosomatic Medicine Rostock University Medical Center Rostock Germany; 2 Deutsches Zentrum für Neurodegenerative Erkrankungen Rostock/Greifswald Rostock Germany; 3 Neurozentrum, Berufsgenossenschaftliches Klinikum Hamburg Hamburg Germany; 4 Fraunhofer Institut für Graphische Datenverarbeitung Rostock Germany

**Keywords:** assistive technology, user-centered design, usability, dementia, smartwatch, mobile phone

## Abstract

**Background:**

Assistive technologies can help people living with dementia maintain their everyday activities. Nevertheless, there is a gap between the potential and use of these materials. Involving future users may help close this gap, but the impact on people with dementia is unclear.

**Objective:**

We aimed to determine if user-centered development of smartwatch-based interventions together with people with dementia is feasible. In addition, we evaluated the extent to which user feedback is plausible and therefore helpful for technological improvements.

**Methods:**

We examined the interactions between smartwatches and people with dementia or people with mild cognitive impairment. All participants were prompted to complete 2 tasks (drinking water and a specific cognitive task). Prompts were triggered using a smartphone as a remote control and were repeated up to 3 times if participants failed to complete a task. Overall, 50% (20/40) of the participants received *regular* prompts, and 50% (20/40) received *intensive* audiovisual prompts to perform everyday tasks. Participants’ reactions were observed remotely via cameras. User feedback was captured via questionnaires, which included topics like usability, design, usefulness, and concerns. The internal consistency of the subscales was calculated. Plausibility was also checked using qualitative approaches.

**Results:**

Participants noted their preferences for particular functions and improvements. Patients struggled with rating using the Likert scale; therefore, we assisted them with completing the questionnaire. Usability (mean 78 out of 100, SD 15.22) and usefulness (mean 9 out of 12) were rated high. The smartwatch design was appealing to most participants (31/40, 76%). Only a few participants (6/40, 15%) were concerned about using the watch. Better usability was associated with better cognition. The observed success and self-rated task comprehension were in agreement for most participants (32/40, 80%). In different qualitative analyses, participants’ responses were, in most cases, plausible. Only 8% (3/40) of the participants were completely unaware of their irregular task performance.

**Conclusions:**

People with dementia can have positive experiences with smartwatches. Most people with dementia provided valuable information. Developing assistive technologies together with people with dementia can help to prioritize the future development of functional and nonfunctional features.

## Introduction

### Background

Dementia is associated with a loss of autonomy and restrictions in coping with everyday tasks [1], which often lead to caregiver burden [2]. There are still no curative treatments for dementia. Assistive technologies (ATs) can help people with dementia maintain their level of everyday activity [3]. To date, digital ATs have not been broadly applied in support and care for people with dementia.

Several systematic reviews regarding digital ATs for people with dementia [4-6] indicate increasing attention being given to wearable devices, for example, smartwatches, which represent the most intimate form of noninvasive ATs. Early digital ATs were aimed at increasing a person’s security by detecting falls and alerting caregivers. Current ATs interact with the wearer and address more than a single domain, for example, reminding the wearer about an event or detecting when the wearer falls [7,8]. The measurement of activities and physiological parameters and the application of user interfaces allow for more flexible support of daily living activities [7,9-12]. Despite the promising potential of ATs, many people with dementia do not use such technologies [13-15]. The reason for this could be that the needs of the target group were not adequately considered, for example, in terms of functional scope or usability of ATs [16]. According to the International Organization for Standardization and the International Electrotechnical Commission 9241-11 standard, usability is defined as the “extent to which a product can be used by specified users to achieve specified goals with effectiveness, efficiency, and satisfaction in a specified context of use” [17].

User-centered innovations address unmet needs and play an important role in breaking barriers and increasing access to ATs [14,18]. Previous studies have shown that the analysis of stakeholder needs, wishes, and values is crucial for sustainable innovations [15,19,20], and focusing on users’ needs potentially prevents ATs from being nonusable or abandoned [13,21,22]. Considering the needs of future users from the beginning of development is mandatory from an ethical and a practical perspective [10,22].

However, placing people with dementia at the center of AT development can be challenging in the following ways: economically—patient involvement may increase the time and cost for organizations involved [23]; empirically—some scholars do not consider accounts of people with dementia to be reliable [24,25]; and ultimately, participation may distress or overwhelm people with dementia [25,26]. In addition, established tools for assessing user experience or usability may be insufficient and difficult to use for people with dementia because their ability to provide insight may be limited [27].

### Objective

We analyzed the usability of a smartwatch application for addressing the needs of people with cognitive impairment based on a user-centered design approach together with people with mild cognitive impairment (MCI) or dementia. This study aimed to contribute to a better understanding of the values and limitations of user involvement in the development of a smartwatch to support people with dementia in their daily lives.

## Methods

### User-Centered Design Framework of the Sensor-Based Individualized Activity Management System for People With Dementia Study

**Figure 1:**
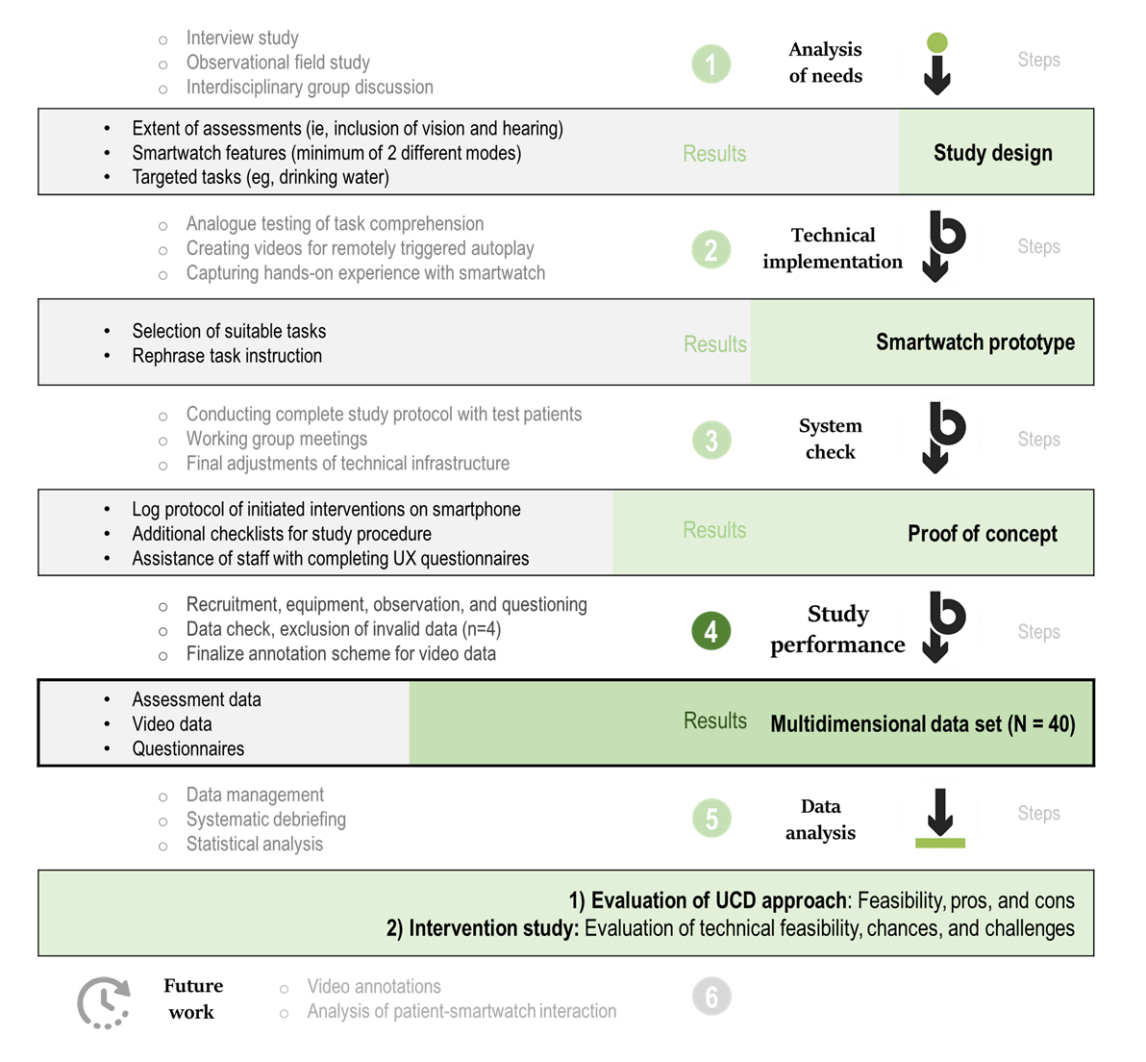
User-centered design framework. Iterative approach to study performance, with the integration of feedback from different stakeholders. Circles indicate different steps. UX: user experience.

The interdisciplinary *Sensor-Based Individualized Activity Management System for People With Dementia* (SAMi) study aimed to develop a mobile assistive device for people with memory impairments to support activities of daily living. The study was planned with a user-centered design approach from the beginning and included stepwise feedback from different stakeholders (Figure 1).

In step 1, we analyzed unmet needs. In a preparatory interview study, we conducted 30 semistructured interviews with stakeholders (people with dementia, health care professionals, and relatives of people with dementia) from a collaborating nursing home located in Pinnow and the geriatric ward of a hospital in Bad Doberan (both are small cities in Northeast Germany). We specifically asked about daily routines and situations in which people with dementia needed help or support. We also addressed the issue of technical assistance, design ideas, and circumstances that promote or hinder the acceptance and adoption of existing and potential future technologies. We applied qualitative content analysis according to Mayring [28] to analyze the material (refer to the study by Köhler et al [29]).

In a parallel, observational field study, we gave a passive smartwatch to 12 people with dementia residing in a nursing home and observed their behavior over a day. This smartwatch passively monitored activity via accelerometers and enabled indoor positioning via Bluetooth. It provided the users with no information except what time of day it was. During the monitoring period, participants’ behavior was annotated in real time by trained observers using the Pocket observer tool (version 3.3; Noldus IT), which included a customized annotation scheme (Multimedia Appendix 1). On the basis of the analysis of needs (step 1 in Figure 1), we conducted an intervention study that aimed to increase certain daily activities by prompting the participants. We have published the results of the interviews elsewhere [29]. The core element of this paper is the results of the intervention study, which represents the final part of the SAMi study.

### Selection of Tasks for the Intervention Study

We decided to prompt participants to engage in 1 task each from the “activity” and “nursing” fields [29]. We implemented short instructions that completely appeared on the smartwatch’s screen. Consequently, we tested different tasks and task comprehension in person with patients from the memory clinic in step 2 (Figure 1). Finally, we implemented a prompt to drink some water (task A; “nursing”) and the “circling bells task” (task B; “activity”) on the smartwatch, as both tasks appeared to be comprehensible and suitable. Instructions for task B explicitly included the instructions to circle bells on a sheet. Further details about step 2 can be found in Multimedia Appendix 2.

### Intervention Study

#### Study Population

The study is registered in ClinicalTrials.gov with the brief title, “SAIN_UMR” (NCT05885620). After a short test phase for system checks (step 3), we started the intervention study (step 4). Participants were recruited from the memory clinic of the Rostock University Medical Center. The inclusion criteria were being aged >50 years, having a diagnosis of MCI or dementia, and having a Mini Mental Status Examination (MMSE) score ≥9 and ≤28 points. The exclusion criteria were presence of clinically relevant impairment of visual acuity or hearing or relevant speech or language impairment. Patients were diagnosed according to international scientific diagnostic criteria, neuropsychological testing (Consortium to Establish a Registry for Alzheimer's Disease [30]), and brain imaging; 78% (31/40) of the patients underwent cerebrospinal fluid analysis. Of the 40 patients, 12 (30%) were diagnosed with MCI [31,32], and 28 (70%) were diagnosed with dementia. Among these 28 patients, 24 (60%) were suspected to be in the Alzheimer spectrum: 18 (45%) with typical Alzheimer disease (AD) [33], 5 (13%) with mixed AD pathology [34,35], and 1 (3%) with atypical AD [33]. Of the 40 participants, 2 (5%) were diagnosed with primary progressive aphasia [36,37], 1 (3%) with a behavioral variant of frontotemporal degeneration [38], and 1 (3%) with Lewy body dementia [35].

Participants underwent clinical and neuropsychological examination—they received standardized examination of visual acuity. Near visual acuity was tested using a standardized optotype card with a decimal scale [39]. Distance acuity was assessed using a standardized eye chart at 4 to 5–m intervals (Oculus, number 4634). Both measurements were uncorrected and, when applicable, corrected with personal glasses. Hearing capabilities were assessed using a tablet-based certified app with a pure-tone threshold test (Mimi Health GmbH). Despite our efforts, we found that a standardized tablet hearing test could not be performed with our participants because they did not tap the button in time. Therefore, we decided to omit the regular test after 20 participants were assessed and did not include the results in our analysis.

As a global score for cognition, we used MMSE [40]. Visual constructive capabilities were assessed using a clock completion test [41] and the Rey complex figure direct copy (Rey Fig Copy) test [42]. Visual attention, processing speed, and task-switching abilities were measured using the Trail Making Test A (TMT-A) and Trail Making Test B (TMT-B) [43]. The results are presented in Table 1. We recruited 44 participants, 4 (9%) of whom had to be excluded. Of the 40 participants, 2 (5%) were diagnosed with subjective cognitive decline, and 1 (3%) had an MMSE score that did not match the inclusion criteria, and the trial procedure of 1 (3%) participant was incorrect because we missed repeating the intervention even though the participant did not complete the task. Finally, for the analysis, we obtained complete data sets from 40 participants. In summary, we included 50% (20/40) women and 50% (20/40) men with a mean age of 75 (SD 6.8; range 58-85) years.

**Table 1:** Demographics and characteristics of the participants^a^. The table includes success scores based on observations and usability scores based on 10 items of the questionnaire.

	Age (y)	MMSE^b^ (points)	Rey Fig Copy^c^	CDT^d^ (score)	TMT-A^e^ (seconds)	TMT-B^f^ (seconds)	Visual acuity–near	Visual acuity–distance	Task A^g^ (score^h^)	Task B^i^ (score^h^)	Sum success score^j^	Usability score
Values, mean (SD)	74.98 (6.68)	23.70 (3.36)	23 (9.09)	2.55 (1.28)	99.10 (66.18)	184.30 (83.56)	0.46 (0.18)	0.69 (0.29)	0.80 (0.38)	0.65 (0.32)	1.45 (0.56)	78.25 (15.22)
Values, median (IQR)	77 (69-80)	25 (22-26)	25.50 (17.5-29)	3 (1-3)	72 (57.5-120.225)	174 (105-239)	0.45 (0.3475-0.5)	0.75 (0.5-0.83)	1 (1-1)	0.50 (0.5-1)	1.50 (1.5-2)	82.50 (68.125-90)
Minimum	58	12	0	1	31	52	0.20	0.13	0	0	0	30
Maximum	85	28	35	6	329	384	1	1.66	1	1	2	100

^a^N=40; women: 20/40, 50%; men: 20/40, 50%; mild cognitive impairment diagnosis: 12/40, 30%; dementia diagnosis: 28/40, 70%; intense intervention mode: 20/40, 50%; regular intervention mode: 20/40, 50%.

^b^MMSE: Mini Mental Status Examination.

^c^Rey Fig Copy: Rey complex figure direct copy.

^d^CDT: Clock Drawing Test (Shulman score).

^e^TMT-A: Trail Making Test A.

^f^TMT-B: Trail Making Test B.

^g^Drinking water.

^h^Score: 0=failure, 0.5=incomplete, and 1=completed.

^i^Circling bells.

^j^Sum of task-A and task-B scores.

**Figure 2:**
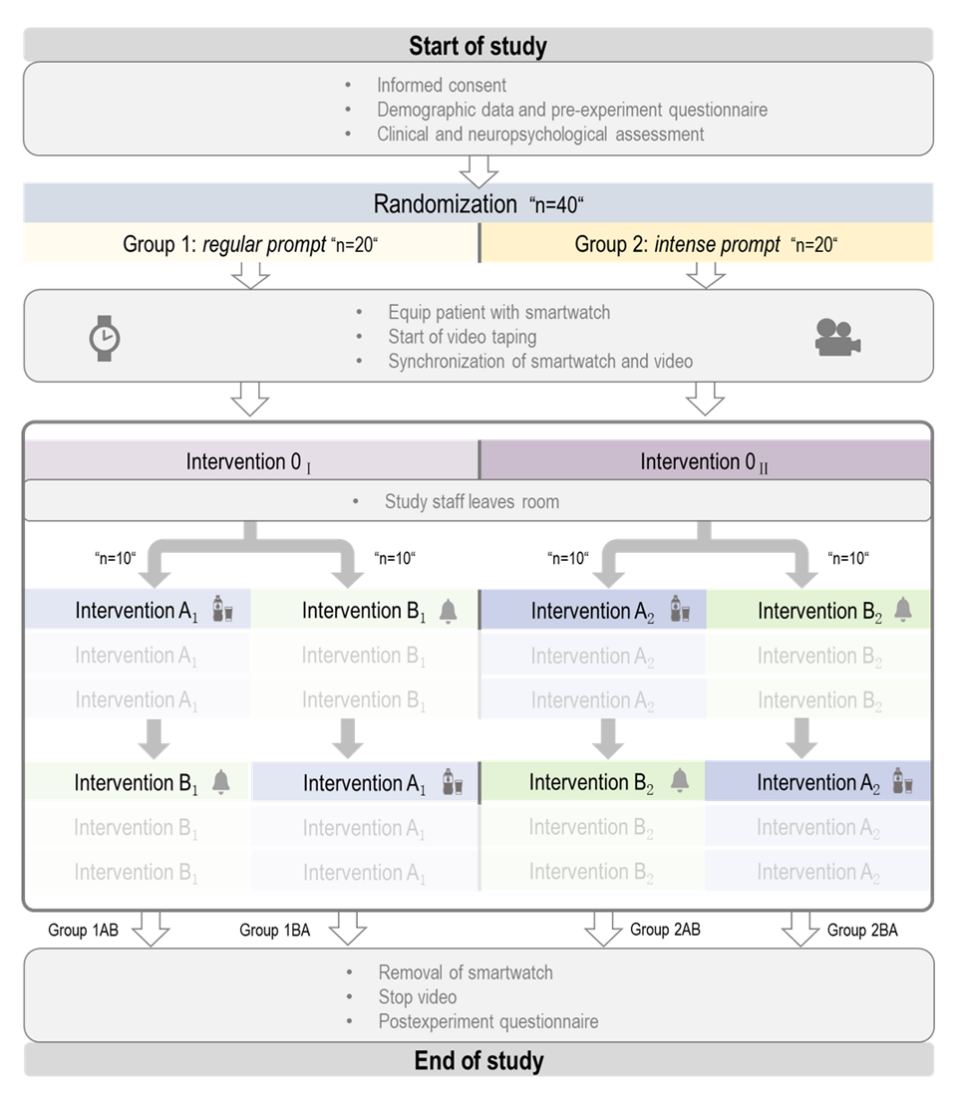
Diagram of the study design.

We conceptualized the observational intervention study based on the feedback obtained from the interview study and the experiences from the field study. Participants received interventions either in the “regular” or “intense” mode, under the observation of 2 cameras. Patients were assigned to one of the groups regardless of their neuropsychological test results. We applied an adaptive randomization procedure to balance the groups regarding participants’ age and sex. Compared to those in the “regular group,” the “intense group” received longer audio and vibration prompts and additional spoken output in response to the written instructions. Finally, a task-related picture appeared on both groups; this picture was animated in the “intense group.” To determine whether the order of the tasks had an effect on their completion, this was tested in both groups additionally. The final result was a study with 2 intervention arms (modus: intense or regular), each consisting of 2 subgroups (order of tasks: AB or BA; Figure 2).

To avoid anxiety, all participants were introduced to the study with a trial prompt initiated by a researcher immediately next to the participant. The trial prompt instructed participants to close their eyes. Then, the participants were instructed to make themselves comfortable and feel free to move within the room. When participants were familiarized with the device and the study procedure, the researchers left the room. After a 5-minute break, the first prompt was triggered remotely. All participants were given both tasks (drinking and circling bells), and prompts were repeated a maximum of 2 times if participants failed to comply. The time delay until repetition was set as 1 minute after the previous prompt.

Participants completed 2 questionnaires. One of the questionnaires captured participants’ previous experiences and affinity with technologies, and the other obtained feedback after wearing the smartwatch under camera observation. The summarized demographic information, test results, and outcomes are listed in Table 1, and additional details are provided in Multimedia Appendix 3.

#### Technological Specifications

**Figure 3:**
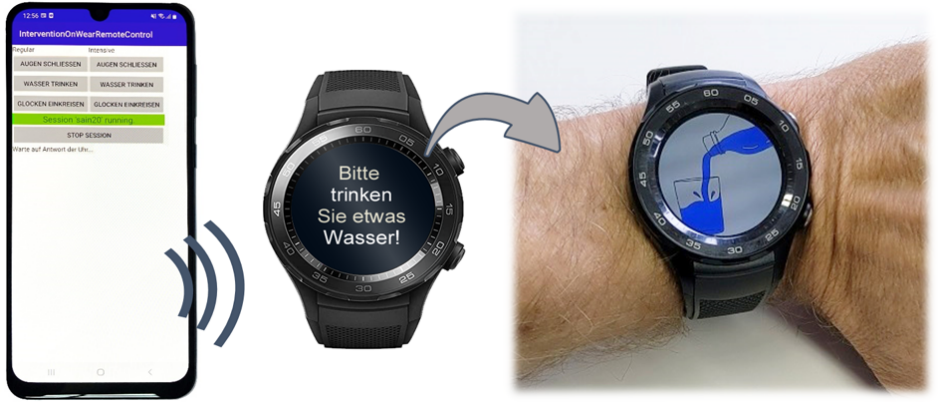
Images and embedding of the applied prototype—smartphone with an app to manually trigger interventions on a watch, which is mounted on the patient’s wrist, showing instructions (in this case, “Bitte trinken Sie etwas Wasser,” which means “please drink some water”).

A Huawei Watch 2 (4G) smartwatch was used. We designed the experiment in a Wizard-of-Oz-setting system using a smartphone as a remote control for the smartwatch (Figure 3). Consequently, we were able to repeat the interventions depending on the remotely observed participant’s compliance (success or failure) or to continue with the next intervention without needing instantaneous detection of behavior via sensors. We set specifications for the smartwatch based on previous experience [44,45] and updated our prototype iteratively; refer to steps 2 and 3 Figure 1. The smartwatch was set to a maximum brightness and volume, with a display size of 1.2 inches. Loudspeakers were limited to 85 dB due to European Union restrictions. In hearing distance, we measured the volume of audio output during interventions to be 56 to 66 dB. The volume varies during signals and speech output from the male voice. We remotely triggered video playback. This approach allowed full control of the font size and audio of the prompts. The length of the videos did not vary between groups. The smartwatch displayed time with the clock hands when no intervention was displayed.

#### Questionnaire

We administered 2 questionnaires to the participants, 1 before and 1 after the intervention. The preintervention questionnaire contained three parts: (1) affinity for technology, based on the Affinity for Technology Interaction scale [46]; (2) personal experiences with technologies; and (3) motivation to participate. It contains 13 items. The postintervention questionnaire was developed according to the Technology Acceptance Model [47], System Usability Scale (SUS) [48], and Technology Usage Inventory [49]. The survey included six subscales: (1) usability, (2) design, (3) perceived usefulness, (4) concerns, (5) realization, and (6) experience during the study. A translated version of the questionnaire with items assigned to the subscales and item coding is available in Multimedia Appendix 4. This questionnaire contains 40 items: 33 closed questions to be answered on a 5-point Likert scale (from strongly agree to strongly disagree), 4 multiple-choice questions, 3 open-ended questions, and 1 opportunity for closing remarks by participants and staff. When we observed participants having difficulty with using the Likert scale, for example, due to alternating positively and negatively worded items, we moderated if necessary. In the moderating questionnaires, we assisted all the respondents while they were completing the questionnaires. If a participant was unsure about what the response scale indicated and whether “agree” or “disagree” indicated their opinion about the particular item, we explained the item in more detail. We also asked participants to review their answers when they accidently skipped questions.

#### Internal Consistency, Usability Score, and Perceived Usefulness

Overall, 3 items of the usability subscale were adapted from the SUS [48], whereas 7 were customized. To obtain a more intuitive score for the usability measurement, we processed the results of our 10-item usability scale analogous to the SUS [48]. In other words, each item was rated 0, 1, 2, 3, or 4 according to the answer on the Likert scale in the following direction: 0 was used for the strongest disagreement and 4 was used for the strongest agreement. The sum of all the scores was subsequently multiplied by 2.5, leading to possible usability scores ranging from 0 to 100. Higher scores indicate better usability.

Answers to the “perceived usefulness” subscale were interpreted similarly. We applied the scale from 0 to 4 for each of the 3 items, resulting in a sum of 0 to 12 responses per participant. Then, the average value of all participants was converted into percentage.

For the subscales related to usability, design, usefulness, and concerns, we calculated the internal consistency as Cronbach α and McDonald ω based on principal factor analysis using Jeffrey’s Amazing Statistics Program (JASP; version 0.16; JASP Team 2021; University of Amsterdam). Missing values were excluded pairwise. The reliability (α and ω) ranges between 0 and 1. Higher values indicate greater agreement among items and suggest that participants’ responses throughout a set of questions were consistent. Cronbach α is a special case of McDonald ω: whereas Cronbach α is based on the assumptions of unidimensionality, equal factor loadings, and uncorrelated errors, whereas McDonald ω accounts for varying factor loadings and error variances, making ω more appropriate to use. Cronbach α also is reported to be consistent with most previous literature.

#### Measure of Success

We observed reactions to the manually triggered interventions via video cameras and rated behavior based on a protocol to decide whether to repeat the intervention. Task A was rated as successful and scored 1 point if the participant drank some water. Task B was rated as successful and scored 1 point if the participant circled something on the worksheet with the pencil. Actions resulting in incomplete task fulfillment were rated with 0.5 points, for example, when a participant went to the table with the worksheet but without using the pen. When no activity that could lead to task fulfillment was initiated, 0 points were assigned. Only the best performance for each task was rated. Repetitions were not scored. The scores for both tasks were summarized, resulting in success values ranging from 0 to 2 points.

### Statistical Analysis

Descriptive statistics, correlations, and 2-tailed *t* tests were performed using JASP (version 0.16). To check for normality, we used the Shapiro-Wilk test. *P* value >.05 was considered to indicate normal distribution. *P* value >.05 in Leven test was considered to be consistent with the equality of variances. For variables that were not normally distributed, we calculated the Spearman rank-order correlation. Otherwise, we used Pearson correlation coefficient. For analysis of the questionnaire items, we used Kendall τ, as the answers on the Likert scale are ordinal data. We chose a significance level of .05, with a corresponding confidence level of 95%.

### Ethical Considerations

Ethics approval was obtained from Rostock Ethics Committee (A 2020-0071). All participants provided written informed consent.

## Results

Demographics, clinical and neuropsychological test results, measures of success, and descriptive measures of the postintervention questionnaire are presented in Table 1 and Multimedia Appendix 5.

### Contributions From Respondents

#### Results Derived From Single-Choice Items

**Figure 4:**
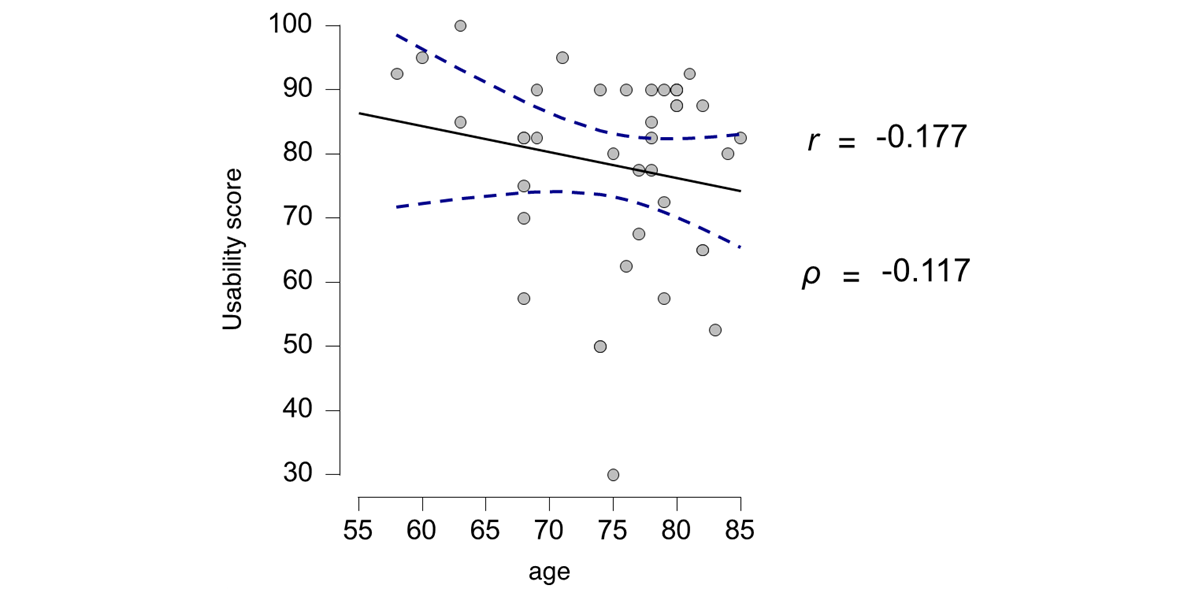
Correlations and regressions between usability and age. The blue lines indicate CIs.

Participants agreed very often with positively worded usability items (Multimedia Appendix 5). Most strongly agreed that they could sense vibration, hear sounds, and recognize visual cues well and had enough time to process the input. Of the 40 participants, 29 (73%) did not find the prompt disruptive. Of the 40 participants, 10 (25%) could not sense the vibration well. Interestingly, of the 40 participants, 23 (58%) agreed with the need to be supported by a technical person. Only one-third (14/40, 35%) were interested in more interactions with the smartwatch. When we processed the usability items analogously to the SUS [48], the mean usability score reached 78.3 (SD 15.4; range 30-100). The score translates as “good” usability score on the original SUS. The score did not significantly differ between men (mean 76.1) and women (mean 80.4; *P*=.39) according to the *t* test. Usability decreased with age, but the effect was not statistically significant (Figure 4).

The design features were satisfactory to many participants. Most (30/40, 75%) found the appearance appealing, and 70% (28/40) found the watch to be properly sized. Further feedback about size was uniformly negative (12/40, 30%), with the watch being criticized for being very large. Of the 40 participants, 37 (93%) stated that the wristband was comfortable to wear. Some participants said that the wristband should be softer (user10), longer (user23), or made from a different material (user17). It was suggested that its clasp be replaced with a magnet clasp (user11). Regarding usefulness, most participants (27/40, 68%) were interested in using the smartwatch frequently and saw a personal benefit in doing so (31/40, 78%). Approximately one-third (13/40, 33%) did not have any idea how the watch could be beneficial. Regarding perceived usefulness of the tool, participants scored an average value of 8.7 (corresponds to 72%), a median value of 9.5 (corresponds to 79%, SD 3.4) Only few were concerned that other people might hear the smartwatches’ announcements (17/40, 43%) or find the watch to be very conspicuous (12/40, 30%). Of the 40 participants, 6 (15%) were concerned about stigmatization because of the watch. Overall, two-thirds of the participants (27/40, 68%) felt a sense of safety while wearing the watch. Of the 40 participants, 5 (13%) were concerned about data protection. Of the 40 participants, 4 (10%) worried about their personal health when using the smartwatch. None of them (0/40, 0%) provided further explanations about these worries. Of the 40 participants, 29 (73%) expressed the wish for a permanent contact person for service. Most (36/40, 90%) felt comfortable with using the watch. Only 8% (3/40) of the participants felt stressed during the trial.

#### Items to Assess Practical Implementation

Practical implementation was investigated using multiple-choice or open-ended questions. We found a preference for less intrusive notifications. A single vibration was favored over multiple vibrations (20>11 checked boxes; 20 persons ticked “single vibrations” and 11 persons ticked the box “multiple vibrations), while a single tone was preferred over ringtones (24>9). Of the 40 participants, 9 (23%) did not indicate their preferred vibration pattern, and 8 (20%) indicated no preferences for ringtones. Of the 40 participants, 24 (60%) thought that instructions should be delivered as spoken output, 20 (50%) preferred written output, and 18 (45%) preferred images. Of the 40 participants, 6 (15%) preferred animations. Of the 40 participants, 17 (43%) indicated only 1 preferred mode of instruction delivery (n=9, 53% chose speech, n=6, 35% chose text, and n=2, 12% chose animation), 18 (45%) preferred a combination of 2 modalities, and 5 (13%) preferred a combination of 3 modalities.

Elaborated feedback was given in the latter part of the questionnaire. Positive feedback was related to the “clear,” “legible,” or “well-arranged” display (user05, user07, user11, user19, and user37); the “easy to understand” audio output (user02, user08, and user41); or the “highly visible timing pointers” (user07). The issues to be optimized included the length of the text display (user04, user27, and user35) and the length of the audio and vibration (user26) files. User07 suggested that “the display should be brighter for a longer time.” The top 5 functions that were chosen for implementation were time display (38/40, 95%), emergency button (34/40, 85%), reminder function (24/40, 60%), telephone option (22/40, 55%), and alarm clock (20/40, 50%). The least useful functions were writing messages (8/40, 20%) or making notes (10/40, 25%).

The answers to the open questions confirmed the preferred functions, especially the need to be supported in navigation (and self-localization) or to be reminded about dates or appointments. In addition, vital parameters were measured. Participants expressed the wish to be directed “within an environment” (user11) and “en route” (user08) and “to find the correct path” (user10) but without further specification. Explicit ideas with points of action were provided for reminders: appointments, daily structure, anniversaries and birthdays, medication, drinking, or reminders of where the house keys are. In terms of vital parameters, people were interested in information about blood pressure, heart rate, sleep, steps, activity, and energy rate. A desired function that was not listed in the questionnaire was the interest in weather forecasting. The extent of overall assistance ranged from “never” (user05) to “for all activities” (user29).

After completing the formal part of the questionnaire, the study staff asked participants who did not respond successfully to the prompts to comment individually. The responses revealed problems with different aspects of the drinking task. User02 said they did not recognize that the watch gave them this instruction. User17 initially could not find the water bottle. User05 did not dare to drink the water provided, and user23 answered that they were not thirsty. We also asked participants for further explanation when they circled the bell on the smartwatch screen instead of the worksheet. Of the 40 participants, 2 (5%) did not think they were supposed to do something in the real world (user33 and user05). User17 indicated that they did not realize that the instructions included the specification, “circle the bells on the sheet.” User34 said they heard the instructions but could not explain why they did not follow it. In addition, user10 stated that they had no idea what to do with the worksheet.

#### Internal Consistency of Subscales

We hypothesized that (1) usability, (2) design factors, (3) perceived usefulness, and (4) concerns play major roles in the user experience. Therefore, we organized the questionnaires into 4 subscales. Internal consistency was determined for each scale (Table 2).

**Table 2:** Internal consistency of subscales.

Subscale	Items, n	McDonald ω (in descending order)	Cronbach α
Usefulness	3	0.81	.81
Concerns	7	0.79	.77
Design	6	0.75	.64
Usability	10	0.65	.64

“Good” internal consistency (ω>0.8) was achieved by the perceived usefulness scale, with concerns and design issues achieving “acceptable” consistency (ω>0.7) and usability features achieving “questionable” levels of internal consistency (ω<0.7). Overall, 3 items proved to be critical (Δ ω>0.1 if the item was excluded) for the internal consistency of their respective scales. Specifically, the items asked about the appropriate watch size, length of the instruction displayed, and desire for frequent use.

### Plausibility of Statements

#### Approach

We evaluated the plausibility of the statements to examine the reliability of the feedback. In this paper, the term, “plausibility,” means “reasonable” or “consistent.” The responses to the items on our questionnaires were neither “right” or “wrong” nor expected by the researchers. Our approach to evaluating plausibility included 3 steps. First, we checked for discrepancies within the questionnaires; second, we compared the questionnaire responses and clinical assessments; and third, we compared the answers from the questionnaires with the observed task performance.

#### Discrepancies Within the Questionnaires

On an intraindividual level, we found very few inconsistencies due to contradictory answers to different items. In the first step, we checked whether positively or negatively worded items were answered consistently, that is, whether the respondent agreed with the positive items and disagreed with the negative items and vice versa. Of the 40 participants, 6 (15%) agreed (“agree” and “strongly agree”) disproportionately with all items (>mean + SD). Of those 6 participants, 5 (83%; user01, user07, user08, user11, and user28) mostly disagreed with the 8 negatively worded items, indicating a positive attitude toward the smartwatch rather than a bias toward positive answers. Only 1 respondent (1/40, 3%; user03) who agreed with the positively worded items also agreed with the negatively worded items. She exhibited an uncritical tendency to confirm statements presented to her (“acquiescence bias”), making it unclear whether her answers truly reflected her point of view or if she misunderstood the questions.

In the second step, we analyzed the participants’ answers regarding the content of their statements. The most implausible answers were found in the questionnaire of user05. She personally completed the questionnaire and indicated high usability (usability score=90) and satisfaction with the design of our smartwatch. Otherwise, she disagreed with the desire to wear the watch in daily life and disagreed with all the questions about usefulness, although she already used a smartwatch in her daily life. She used open-ended questions, for example, to suggest a smaller size but did not explain the reason for indicating high usability and satisfaction with the app design despite her concerns. She answered the question for desired domains of support with “not at all” after canceling her initial answer, “always.” Fewer inconsistencies were found in the questionnaires of user26. She preferred single vibrations and short ringtones instead of multiple or repeated vibrations and longer ringtones but recommended prolonged vibration and anticipated possible difficulties in hearing the sounds of the smartwatch for people with hearing impairment. In addition, user22 did not clearly indicate his preference regarding sounds. He selected both sound options even though they were mutually exclusive.

At a group level, it appeared implausible that less obtrusive notifications regarding audio (24>9) and vibration patterns (20>11) were favored, whereas speech output was a desired characteristic for most participants (24/40, 60%).

#### Correlations Between the Questionnaire and Assessment Data

When asked to mention situations in their daily lives that should be supported by a smartwatch, 11% (3/28) of the people with dementia stated no need for support (refer to the Items to Assess Practical Implementation section), although their caregivers reported a need for help that led to the diagnosis of dementia. This implies that these participants have lost insight into their functioning in daily life, limiting their reliability in naming domains for necessary support. Otherwise, they also anticipated a decline, as indicated by the added terms, “not yet,” “not to date,” and “not at present.” In addition, there was a discrepancy between the need for support for people with dementia and the severity of their impairment. One of the patients with MCI (1/12, 8%; user29) felt the need for support “for all activities.”

**Figure 5:**
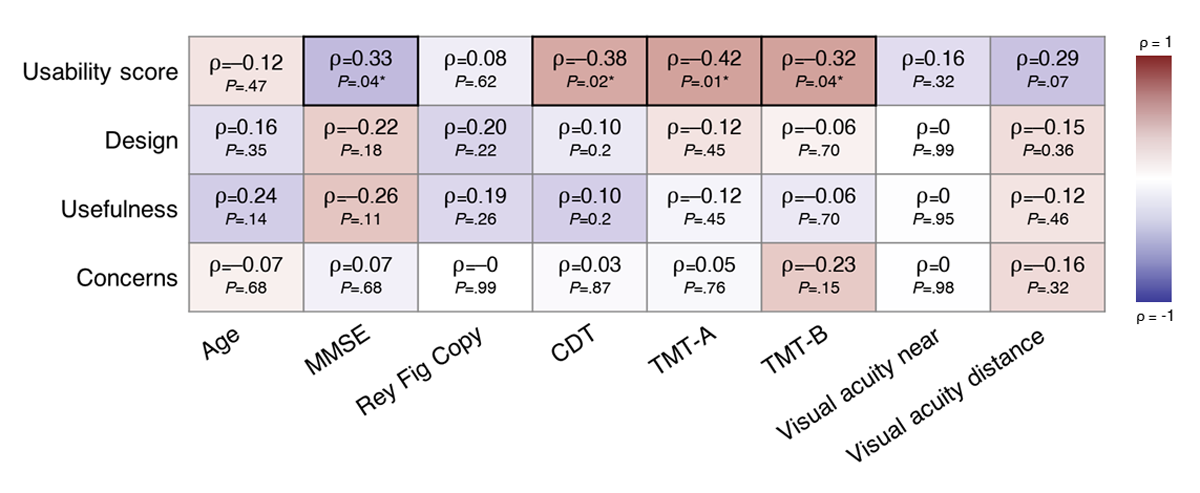
Heat map of Spearman correlation coefficients (ρ) and *P* values for the subscales and assessment data. Asterisks indicate statistical significance. CDT: Clock Drawing Test (Shulman score); MMSE: Mini Mental Status Examination; Rey Fig Copy: Rey complex figure direct copy; TMT-A: Trail Making Test A; TMT-B: Trail Making Test B.

We also correlated the neuropsychological test results with the results from the subscales of the questionnaire. A better usability score (the higher the score is, the more usable the smartwatch appears to be) was significantly correlated with better cognition, as suggested by a positive association with the MMSE score (*P*=.04) and negative associations with the Shulman score on the clock completion test (*P*=.02) and time on the TMT-A (*P*=.01) and TMT-B (*P*=.04; Figure 5).

This confirmed our hypothesis that neuropsychological performance is associated with usability. The 3 other subscales did not correlate significantly with any neuropsychological test. Regarding sex, the 4 subscales did not significantly differ between the 2 groups.

We also checked for conformity of single items with the clinical test results. We correlated item 5 in scale 1 (recognizability of visual prompts on the smartwatch screen) with the visual acuity test results and found no relevant correlation (τ_Visual acuity N_ –0.24, *P*=.09; τ_Visual acuity D_ –0.16, *P*=.23) Only 5% (2/40) of the participants disagreed with good visibility. Both had a visual acuity below average. The 2 items focusing on latency, asking whether participants had sufficient time to notice that the watch notified them and whether instructions were shown for an adequate time, were correlated with the results of the TMT-A and TMT-B. In addition, no correlations were found (ρ<0.3; τ<0.2; *P*>.05, respectively).

#### Questionnaires and Observed Task Performance

At the group level, single vibrations and less intrusive audio were preferred (refer to the Items to Assess Practical Implementation section). Regarding completion rates, participants in the “intense” group were more often successful. Here, we can see a discrepancy between the desire for less intrusive signals and better outcomes in participants with more intense intrusiveness.

**Figure 6:**
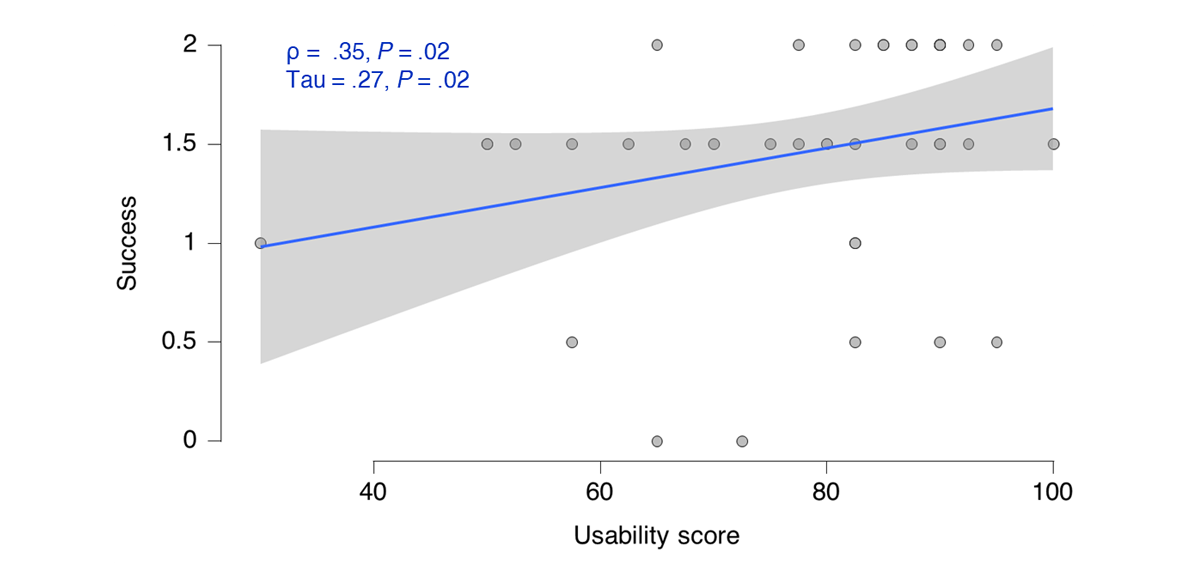
Association between success and usability. Success was rated based on observations, and usability was self-rated by participants via questionnaire.

We correlated the success (for the score calculation, refer to the Measure of Success section) with the usability score (Figure 6). Here, we found a significant positive correlation between the 2 measures; however, the effect size was small to moderate (τ=0.27). The higher the usability was, the more successful the participant was.

**Figure 7:**
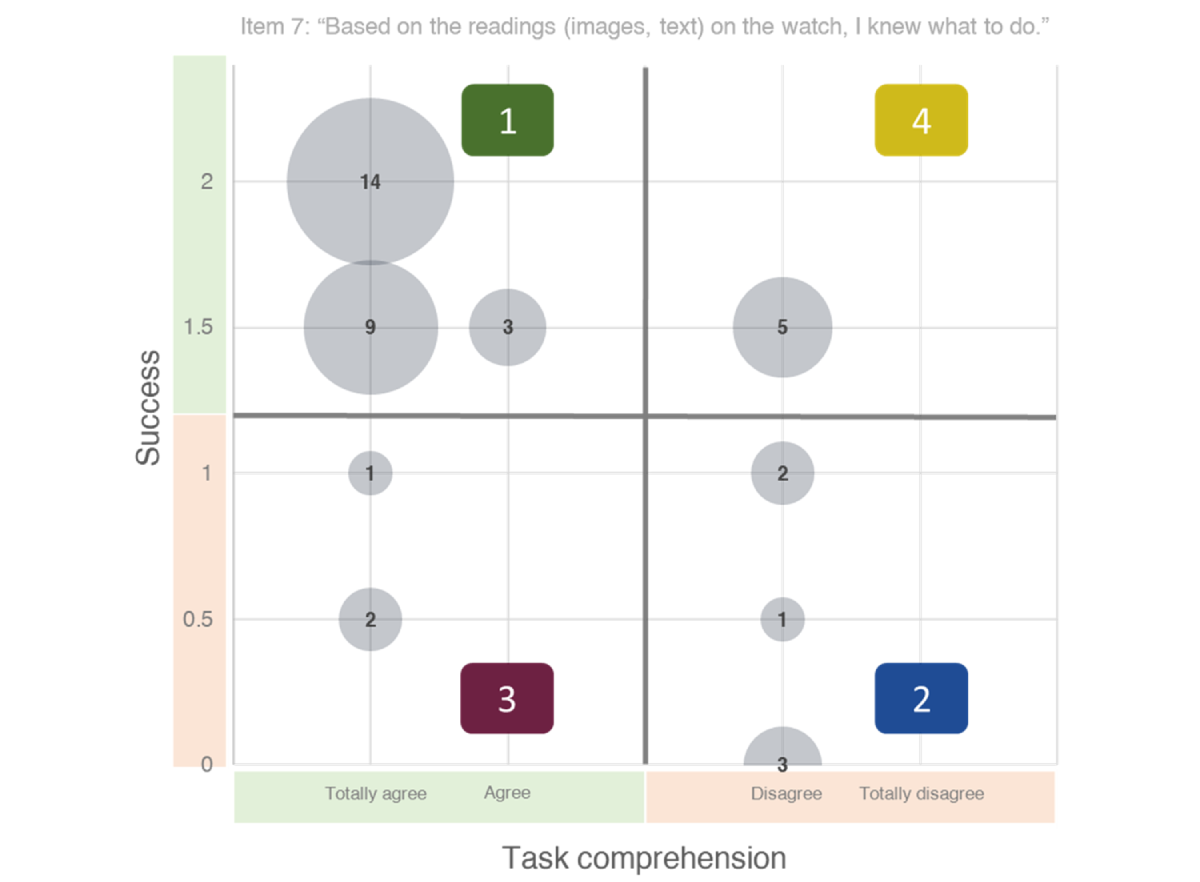
Matrix of self-evaluations and observed task performance. The number in gray circles corresponds to the number of participants. Cluster 1: successful participants with concordant self-reflection, cluster 2: unsuccessful participants with concordant self-reflection, cluster 3: unsuccessful participants with disconcordant self-reflection, and cluster 4: successful participants with disconcordant self-reflection.

We also checked for concordance between self-evaluations and observed behaviors at the individual level by investigating the overlap between success and specific items from the questionnaire. We divided each data set into 2 groups, resulting in 4 clusters (Figure 7). Regarding self-reflection, we distinguished between participants who agreed (clusters 1 and 3) and those who disagreed (clusters 2 and 4) based on item 7 of the usability scale, which assessed task comprehension. Regarding success, we distinguished between participants who completed no more than 1 task (clusters 2 and 3) and others (clusters 1 and 4).

We could see the concordance of self-reflection and observation in most participants (cluster 1: 26/40, 65% and cluster 2: 6/40, 15%). Of those people with appropriate self-reflection, most were successful (14/40, 35%) or almost completely successful (9/40, 23%). They correctly stated that they knew what they had to do. All the 12 people who were not completely successful had trouble with task B (circling bells). Of the 12 participants, 11 (92%) circled the bell on the smartwatch screen and 1 (8%) with the finger on the worksheet. In addition, participants from cluster 2, who were not able to complete >1 task, concordantly disagreed with good task comprehension. Interestingly, no one, including participants who did not attempt either task, strongly disagreed. Few (8/40, 20%) participants showed a discrepancy between self-reports and observations (clusters 3 and 4). We identified 3 possible reasons for the deviation in people in cluster 3. As shown previously, user05 was suspected to have had trouble in completing the questionnaire. We found inconsistencies within their questionnaire and suspected misinterpretation of questions or rating scales. Moreover, memory impairment might limit self-reflection. When completing the questionnaire, user02 indicated that she preferred to have had it handed out beforehand because she could not remember specific details (eg, vibration). User02 was diagnosed with AD dementia and had a score of 22 on the MMSE.

Finally, we found that incomplete or irregular fulfillment of tasks was not recognized by the concerned participants. Except for 1 participant (1/8, 13%; user02), all participants from clusters 3 and 4 completed task B and circled the bell image that appeared on the smartwatch screen. The individuals in cluster 3 felt that their understanding of the task was good, as they felt that they solved the task, even if they did not perform the task according to the researchers’ success protocol. This clear discrepancy between self-reflection and observation might indicate a loss of insight into their abilities and behavior. User33 explained that he did not consider using objects from the real world. User33 and user05 did not drink when prompted. Both belong to cluster 3. It is conceivable that some participants did not expect any actions involving their environment. Otherwise, all people in cluster 4 (5/5, 100%) managed the drinking task and circled the bell on the screen. It is unclear why they disagreed with task comprehension.

### Summary

This paper describes an interdisciplinary study to identify the needs regarding and analyze the effects of multimodal interventions using auditory, haptic, and visual information provided by a smartwatch.

We hypothesized that perceived usability, design, usefulness, and concerns would influence the user experience with our smartwatch and, consequently, included those topics using our questionnaire. We saw interest and commitment from the participants. Some participants struggled with the Likert scale, which might be avoided by guiding them or modifying the questionnaire. Usability was evaluated using complementary approaches, including questionnaire-derived measures and observational ratings of success based on completion rates. Usability, quantified through a 10-item score, reached a mean of 78 (maximum possible score=100). This finding is consistent with the remote observations: 35% (14/40) of the participants were able to complete both tasks, and 50% (20/40) of the participants solved one of both tasks. Only 15% (6/40) of the participants were unable to complete either task. Nonetheless, more than half of the respondents (23/40, 58%) thought they would need the support of a tech-savvy person to use the watch in the long term, and many (29/40, 73%) expressed the wish for a permanent contact person for maintenance. Only one-third (14/40, 35%) wanted more interaction with the smartwatch. The design was appealing to most participants, even though feedback about the size of the watch was primarily negative, with participants expressing that they felt the watch was very large. Various elements of the hardware have received suggestions for improvement. Perceived usefulness was rated high. Overall, two-thirds of the participants (27/40, 68%) had a sense of safety with the watch, and one-third of the participants (13/40, 33%) did not believe that the smartwatch would be beneficial. Only few respondents were concerned about using the watch. We also assessed nonfunctional requirements. We observed a preference for less intrusive notifications on the one hand and a desire for voice output on the other. Sophisticated feedback was provided about the wristband and the display latencies. Several specific use cases for possible reminder functions were mentioned. The internal consistency of the subscales varied between “questionable” and “good.” Lower cognitive ability was associated with lower usability.

We evaluated the plausibility of the feedback by checking for discrepancies. Only very few intraindividual inconsistencies were found within the questionnaires. There were no signs of a general acquiescence bias. When comparing self-rated task comprehension and observations, we observed concordance in 80% (32/40) of the participants. Mild deviation of self-reflection and task completion was observed in 13% (5/40) of the participants, and strong deviation was observed in 8% (3/40) of the participants. Participants seemed to lack insight into incomplete or irregular task performance. At the group level, we observed conflicting findings related to intrusiveness. Preferences for less audio and vibration conflicted with the desire for voice output, and more intrusiveness led to more successful task completion.

## Discussion

### Indications of Bias

Participants provided very favorable feedback. It is possible that our participants were not overly critical because they had little experience with better-designed, equivalent technologies. Older adults have a limited understanding of the potential implications for their privacy [50]. High usability could also be a sign of recruitment bias. Our recruitment strategy was not biased toward people who are interested in technology, as we invited all participants from the memory clinic. Previous experience with mobile technologies was not necessary. However, interest in the research topic is a major driver of participation; therefore, one must assume that participants were interested in technologies at least slightly.

We cannot rule out acquiescence bias, a tendency to choose the first option or a tendency to choose positive response options (agreement). We controlled for this bias by considering answers to inverted items: most of those items were consistently answered. This makes agreement bias unlikely. High levels of agreement possibly reflect a courtesy bias: the tendency to understate dissatisfaction or challenges with a system, driven by politeness. It is possible that respondents who anticipated positive feedback would be more desirable to researchers than those who provided negative feedback. At a metalevel, this poses a problem in participatory research and should be further investigated. When treated as coresearchers, people may be confused with different roles [51] and transform their commitment to science and innovations into less critical feedback in return for their empowerment. The participants were also reported to be motivated by family members and researchers [25]. Uncertainties regarding participants’ roles in participatory research have already been noted [26]. Otherwise, the participants who actively provide feedback or even participate as coresearchers are motivated to make a difference and transfer scientific results into practice [52]. Regarding ATs for people with dementia, professional researchers and coresearchers share the same need for technology transfer. Neither would benefit from spending resources for the development of unsuitable technologies as a result of uncritical evaluation. Transparency and clarification of expectations, roles, and goals could help resolve this conflict.

### Rating of Usability and Review of Internal Consistency

Our calculated usability score cannot be compared directly with other scores resulting from SUS ratings, as we deviated from the original SUS by 7 items [48]. However, regarding the mean and SD, our score is similar to other SUS scores [53,54]. As described previously, usability decreases with age [53] and poorer cognition. We presented data about the internal consistency of the subscales of our questionnaire, with McDonald ω being between 0.65 and 0.81. These findings are consistent with those of previous studies about the internal consistency of usability questionnaires administered to older adults or people with cognitive impairment [54,55]; however, we found only a small number of studies regarding this topic.

### Feasibility and Significance of User-Centered Design

There is an ongoing discussion about the extent to which people with dementia can be involved in research and in the development of ATs [20,25]. The ambiguous nature of participation has been examined in several recent reviews. Brett et al [23] showed many positive aspects of patient and public involvement in general; however, they also stressed that the outcomes were found more randomly than methodically. Kowe et al [26] summarized many advantages and disadvantages of participatory dementia research for researchers. Fischer et al [20] discovered positive and negative effects of involvement of older users in technology design but were unable to determine its impact on technology adoption and acceptance. However, Bethell et al [56] focused on engagement of patients with dementia but could not determine its impact on the research process or outcomes. All of them concluded that more evidence is needed to illustrate the impacts on the involved parties. Therefore, we aimed to determine the feasibility of user-centered development of smartwatch-based interventions for people with dementia.

We based our analysis on 3 aspects: study implementation, intervention outcome (success metrics), and qualitative measures (user experience and perceived usefulness). Regarding recruitment, we had no difficulty in identifying and enrolling suitable study participants. We found participants to be interested and dedicated to this field of research. We had only a small amount of missing data. This indicates that our study addressed a problem relevant to the target group. As we wanted to evaluate the feasibility of the user-centered design approach, we do not discuss about its technical feasibility in this paper. Regarding intervention outcome, we could see that 80% (32/40) of the participants were able to completely solve task A and 40% (16/40) of the participants completely solved task B; a further 50% (20/40) of the participants completed task B at least partially. As these values are study specific and there are no values in the literature for comparison, it is difficult to determine what was to be expected. Many researchers portray older adults as technologically illiterate [20]. Many people in the target group had significant visual, hearing, and tactile impairments [57,58]; therefore, it was not clear whether a smartwatch would even be able to reach the threshold required to gain the attention and task understanding of these people. In this regard, the task completion rate in our study seems to be high. This high rate could also be explained by our interventions being common tasks requiring little effort. In addition, our tasks did not require participants to directly interact with the smartwatch.

Finally, regarding user experience, we received very positive feedback. Most participants felt comfortable with using the watch and would be willing to participate again in the study. Overall, three-fourth of the participants (31/40, 78%) could imagine having a personal benefit from using the watch, which is indicative of a high level of perceived usefulness. On the basis of the evaluation of these 3 aspects, we definitively consider the feasibility as given, but note that user involvement is more resource intensive [20].

The involvement of various stakeholders represents an additional expense [24]. On the one hand, it takes more time to conduct and, above all, evaluate interviews. On the other hand, the creation of suitable questionnaires is methodologically challenging. The scope (number of questions), the number of response levels, or the alternation of positively and negatively worded questions [59] are essential aspects that need to be considered [60]. The iterative approach [20] means that substeps and project goals cannot be defined and planned from the outset. Instead, interim evaluations and adjustments are necessary, which require consistent project management and effective team communication. In our case, the many years of expertise in this area and the commitment of the study staff contributed to our success, as did the long-term, third-party funded financial support over 3 years.

### Plausibility Analyses

The second objective was to evaluate the extent to which user feedback from people with dementia regarding ATs is plausible. Our approach to evaluate plausibility included three steps: (1) analysis of discrepancies within questionnaires, (2) comparison of questionnaire responses and clinical assessments, and (3) comparison of questionnaires and observations.

Individual-based analyses revealed only isolated inconsistencies, and group-based analyses revealed inconsistencies that may indicate mutually exclusive needs. Participants showed a wish for less obtrusive notifications and dislike toward a watch that was very conspicuous but also stated a wish for speech output. This shows that there will be no technology that can address all needs equally. There can be conflicting needs both interindividually and intraindividually. This inconsistency in the statements is not due to cognitive deficits but reflects ambivalent attitudes that exist in all people to a greater or lesser extent [29]. We emphasize that unreliability in certain individual cases should not be a reason to prevent the whole group of patients with dementia from participating via self-reports [51].

Our results showed a moderate correlation between perceived usability and cognition (Figure 5). These findings were previously described [53] and confirmed our hypothesis that neuropsychological performance is associated with usability.

In contrast to previous studies [61], we observed high rates of overlap between self-reflection and success metrics such as completion rates. The concordance was evident in both directions. Objectively successful participants mostly indicated a good understanding of the task, whereas less successful participants disagreed with this statement. Only 8% (3/40) of the participants showed a clear discrepancy in the sense that they did not recognize their failure. Overall, 18% (7/40) of the participants did not recognize incomplete or irregular task fulfillment.

The answers were generally plausible and, in part, even elaborated.

### Added Value of Our Study

This study is one of the few investigations of the interaction of people with dementia with an interactive smartwatch. Particularly noteworthy is the detailed clinical characterization of the study participants and the exact description of the technical features of the smartwatch interventions. The publication of the results of the survey about the wishes and needs of people with proven memory impairment regarding a smartwatch alone is valuable, even if the sample size of 40 individuals is not particularly large. Several specific use cases were mentioned, and new ideas for wishful smartwatch features were suggested.

Although there are numerous recommendations regarding the design of user interfaces of new technologies, for example, user interface design, these have rarely been tested in a scientific setting in practice on older adults or people with memory impairment. This target group is extremely heterogeneous, and it must be assumed that general recommendations are of limited value [20]. Therefore, it was not clear whether a smartwatch would even be able to reach the threshold required to gain the attention and task understanding of these people.

Most studies of smartwatches for patients with dementia have no interactive claim but, instead, use passive monitoring [62]. We could only find 3 studies that used an interactive smartwatch for people with dementia to support activities of daily life [8,63,64]. König et al [8] designed a system in which a smartwatch was embedded into an infrastructure with tablets and a web platform to evaluate usability in a 3-month trial. The authors did not observe significant changes in the quantitative measurements. They did not assess single interactions. Thorpe et al [63] tested smartwatches capable of scheduling, navigation, communication, and orientation using an off-the-shelf wearable device in 5 participants. Personalization and familiarity appeared to be key drivers of smartwatch adoption. The task completion rates varied greatly among the participants, as in our study. McCarron et al [64] tested a face recognition application for smartwatches paired with smartphones to improve the quality of social interactions and quality of life among people with dementia. The authors had no trouble with study implementation and reported no problems with feasibility. Furthermore, they found no overall impact on the quality of life of the 48 participants when they used the smartwatch for 6 months.

Compared to other studies, our study has a sound sample size [12,15] and represents great added value, as it expands the small body of scientific literature in this specific area.

At a metalevel, this study is important because it highlights the practical implications of user-centered design in the development of novel technologies together with people with cognitive impairment, for example with giving precise recommendations for usability questionnaires for people with dementia, (Multimedia Appendix 6).

### Limitations

Specific reasons for discomfort or concerns could not be captured using the questionnaire. In addition, contradictory information regarding the preferred intrusiveness of smartwatch notifications could not be resolved in this study. Some other questions remain open. What authority do we ascribe to technical devices? In this study, 1 participant (1/40, 3%) commented that they would not willingly drink the water that was provided even though the smartwatch prompted them to drink it. Another user stated that they were not thirsty. Such context-related information cannot be measured by sensors. If we want users to actively interact with a device, then questions about the immersive character arise. How should users know when they should interact with the smartwatch and when they should interact with their environment? The smartwatch specifications that we applied in our study seem to match the needs of users. Minor adjustments should focus on optimizing display latencies and wristbands. Future studies should implement more functions and test those functions in practice with more participants in real-world scenarios in the long term.

### Conclusions

To determine whether user-centered design featuring people with cognitive impairment in the development of digital assistive devices is worthwhile, one needs to measure the utility of the developed technology. This can be accomplished by evaluating the usability, usefulness, and success metrics. All 3 approaches provide unique challenges because the available measuring tools may be inapplicable for the technology at hand or for the group of potential users. On the basis of the results of our study, we derived specific recommendations for questionnaires for people with dementia. Measures of internal consistency should not be overrated.

To improve technology adoption, the concept of “technical dyads” might be useful: each user is assigned to a person who is willing and capable of adjusting and maintaining devices for users who are technically inexperienced. This would be consistent with the needs of our participants, who expressed the wish for a permanent contact person for service. This wish also confirms the results that have already been found by others [10].

Our study is one of few studies that examined an interactive smartwatch for people with dementia. Although there are some methodological challenges for such studies, we and others have shown that both the inclusion of future users and the use of smartwatches by people with dementia are possible.

## Appendix

Multimedia Appendix 1 Sensor-Based Individualized Activity Management System for People With Dementia observational field study.

Multimedia Appendix 2 In-person testing of intervention tasks.

Multimedia Appendix 3 Demographics and test results.

Multimedia Appendix 4 Postexperiment questionnaire used in the intervention study.

Multimedia Appendix 5 Descriptive results of the applied questionnaire.

Multimedia Appendix 6 Recommendations for usability questionnaires for people with dementia.

## Abbreviations

AD: Alzheimer disease
AT: assistive technology
JASP: Jeffrey’s Amazing Statistics Program
MCI: mild cognitive impairment
MMSE: Mini Mental Status Examination
SAMi: Sensor-Based Individualized Activity Management System for People With Dementia
SUS: System Usability Scale
TMT-A: Trail Making Test A
TMT-B: Trail Making Test B

## Data Availability

The data sets used and analyzed during this study are available from the corresponding author upon reasonable request.
