# Supplementary material for: Smartwatch-Based Interventions for People With Dementia: User-Centered Design Approach
Source: JMIR Aging. 2024 Jun 7;7:e50107. doi: 10.2196/50107 (PMC11193079; doi:10.2196/50107)
Supplement: Multimedia Appendix 1 [file aging_v7i1e50107_app1.pdf]

Supplement

1) SAMi - observational field study: 09/2019 - 02/2020

Objective:

- explore options for suitable interventions that provide meaningful assistance for PwD[1]
- test feasibility of sensor-based recognition of place, posture and activities of residents

Methods:

- field study with PwD living in one nursing home, map of ground floor shown below
- participants wore smartwatches to measure activity (accelerometry)
- indoor positioning was realized via Bluetooth low energy (via smartwatch)
- live observation and annotation of behavior was performed from trained study staff in parallel to sensor recording
- observed domains: place, posture, activity, behaviors of special interest, e.g. disorientation, falls, request for help

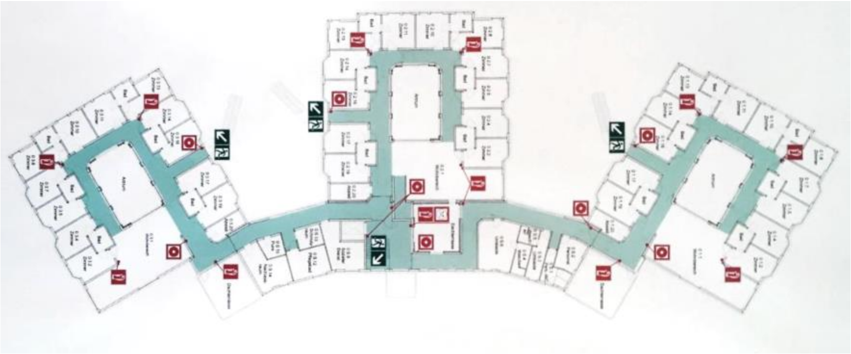

Figure 1s: Ground floor of nursing home. Residents live in single rooms, arranged in circles of three living areas. Each living area contains a kitchen and a living room and is colored with a different wall color.

Place	Posture	Activity	Others	Not observed
Patient's room ■	Lying ■	ADL ■	Spatial disorientation ▼	Not observable ■
Restroom ■	Sitting ■	Purposeful activity ■	Other disorientation ▼	
Common sitting room ■	Standing ■	Agitation ■	Fall ▼	
Hallway ■	Walking ■	Inactivity ■	Caregiver assistance ▼	
POI (Point of interest) 15 different specified: e.g. Elevator, Emergency exit ■	Other posture ■	Sleep ■	Request for help ▼	
	Posture unknown ■	Unknown activity ■	Manipulation at device ▼	

Figure 2s: Annotation scheme of observation study as part of analysis of needs. Black squares indicate state events, with start, duration and end; black arrows indicate point events.

Results:

- N=12 PwD included
- Age: 74-95 years
- 2 male/10 female
- moderate to severe stages of dementia (MMSE 5-18 points, ∅11,2)
- ~ 302 hours of observational data from natural occurring behavior during daytime (8AM-4PM)

---

[1]Abbreviations: PwD: person with dementia   MMSE: Mini-Mental-Status-Examination
