# Supplementary material for: Smartwatch-Based Interventions for People With Dementia: User-Centered Design Approach
Source: JMIR Aging. 2024 Jun 7;7:e50107. doi: 10.2196/50107 (PMC11193079; doi:10.2196/50107)
Supplement: Multimedia Appendix 2 [file aging_v7i1e50107_app2.pdf]

Supplement

2) In-person testing of intervention tasks

In-person testing of different tasks was done prior to technical realization in the intervention study. The testing was performed without technical devices. Verbal instructions were always given in the exact same way from study staff.

We tested different activation tasks, e.g. solving a four-piece jigsaw puzzle or circling symbols on a worksheet adapted from Bells test with ten different patients, respectively. Mean age of all 20 participants was 73 years, mean MMSE result 25.3 points[1]. Nine of ten patients immediately solved the puzzle. Task completion was done within 15 seconds (mean) whereas the bells took longer (mean: 30 seconds) and were only completed correctly from seven of ten patients. The bells seemed to be more challenging. This was based on the observation, that task completion took longer and success rates were lower. In line with the idea of activation, we decided to implement the bells task on the smartwatch.

Test Intervention: complete jigsaw puzzle

	#1	#2	#3	#4	#5	#6	#7	#8	#9	#10	mean
**age**	80	66	82	76	76	62	87	78	76	71	75,4
**sex**	f	f	f	f	m	m	f	m	f	f	3m
**MMSE**	26	29	23	27	29	27	21	23	28	26	25,9
**time to completion [s]**	137*	8	9	16	11	20	24	23	13	7	14,6
**success x/4**	0	4	4	4	4	4	4	4	4	4	3,6

* resigned

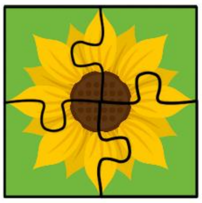

Test Intervention: circle bells on worksheet

	#11	#12	#13	#14	#15	#16	#17	#18	#19	#20	mean
**age**	81	65	66	51	69	75	76	66	77	75	70,1
**sex**	f	f	m	m	f	f	f	f	m	m	4m
**MMSE**	18	23	22	30	27	23	29	28	27	19	24,6
**time to completion [s]**	40	43	60	35	20	24	25	16	25	8	29,6
**success x/5**	3	1*	5	5	5	5	5	5	4	5	4,7

* + 3 wrong symbols circled

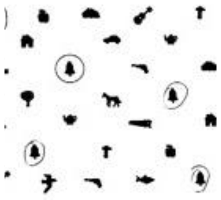

[1] sex: f= female, m= male
