# Supplementary material for: Smartwatch-Based Interventions for People With Dementia: User-Centered Design Approach
Source: JMIR Aging. 2024 Jun 7;7:e50107. doi: 10.2196/50107 (PMC11193079; doi:10.2196/50107)
Supplement: Multimedia Appendix 3 [file aging_v7i1e50107_app3.pdf]

Supplement: 3) Demographics and test results

group	participant	sex	diagnosis	age [years]	MMSE [points]	Rey Fig Copy	CDT [score]	TMT-A [sec]	TMT-B [sec]	Visual acuity N	Visual acuity D	task A [score]	task B [score]	sum success	Usability score
intense	user01	m	MCI	82	27	20	1	73	210	0.5	0.91	1	1	2	87.5
	user03	f	dementia	68	23	22	4	174	384	0.2	0.125	1	1	2	82.5
	user08	f	dementia	80	18	11	3	81	NF	0.5*	0.83*	1	1	2	90
	user12	m	dementia	79	27	27	1	112	NF	0.4*	0.68*	1	1	2	90
	user14	m	MCI	78	27	33	3	45	105	0.67*	0.3*	1	1	2	77.5
	user19	f	MCI	63	28	33	1	37	67	0.5*	0.83*	1	1	2	85
	user25	f	dementia	82	21	2.5	4	282	NF	0.2	0.33	1	1	2	65
	user29	f	MCI	69	28	28	1	67	104	0.5*	1*	1	1	2	90
	user32	f	dementia	78	20	20	3	63	NF	0.3*	0.5*	1	1	2	85
	user37	m	dementia	74	24	33	1	31	85	0.67*	0.83*	1	1	2	90
	user10	f	dementia	84	23	0.5	5	240	NF	0.4*	0.62*	1	0.5	1.5	80
	user15	m	dementia	74	25	17.5	3	135	NF	0.4	0.25*	1	0.5	1.5	50
	user18	m	dementia	80	25	35	1	71	188	0.4*	0.62	1	0.5	1.5	87.5
	user31	f	dementia	68	25	30	1	59	193	0.5*	1*	1	0.5	1.5	75
	user34	f	dementia	77	22	28	1	37	97	0.3*	0.41*	1	0.5	1.5	67.5
	user36	f	dementia	75	26	25	2	66	NF	0.2*	0.5*	1	0.5	1.5	80
	user38	m	MCI	76	25	29	2	102	345	0.4*	0.75	1	0.5	1.5	90
	user39	m	dementia	80	22	17	4	104	NF	0.2	0.75	1	0.5	1.5	90
	user44	m	dementia	69	23	25.5	3	101	NF	0.33	0.5	1	0.5	1.5	82.5
	user22	m	dementia	75	22	NA	4	329	NF	0.4*	0.27*	0	1	1	30
intense	Mean	10m /10f	5 MCI / 15 dementia	75.6	24.1	23.0	2.4	110.5	177.8	0.4	0.6	1.0	0.8	1.7	78.8
	Median			76.5	24.5	25.5	2.5	77.0	146.5	0.4	0.6	1.0	1.0	1.8	83.8
	Minimum			63.0	18.0	0.5	1.0	31.0	67.0	0.2	0.1	0.0	0.5	1.0	30.0
	Maximum			84.0	28.0	35.0	5.0	329.0	384.0	0.7	1.0	1.0	1.0	2.0	90.0
regular	user16	m	dementia	71	26	28.5	2	41	52	0.3*	0.33*	1	1	2	95
	user24	f	dementia	81	23	32	2	59	239	0.8	1.66	1	1	2	92.5
	user28	f	dementia	78	27	27	2	47	174	0.67*	1*	1	1	2	90
	user41	f	dementia	80	25	27	1	90	187	0.4*	0.27*	1	1	2	87.5
	user04	m	MCI	68	26	25.5	2	67	245	0.2	0.83	1	0.5	1.5	57.5
	user07	m	dementia	77	22	0	4	127	NF	0.4*	0.83*	1	0.5	1.5	77.5
	user11	f	MCI	63	27	24	2	69	150	0.4*	0.83*	1	0.5	1.5	100
	user17	f	dementia	58	24	13.5	3	67	277	0.5*	0.62	1	0.5	1.5	92.5
	user26	f	MCI	76	25	35	2	66	142	0.8	1.11*	1	0.5	1.5	62.5
	user27	m	dementia	68	23	27	1	155	154	0.5	0.75	1	0.5	1.5	70
	user35	f	MCI	83	25	25	3	53	173	0.67*	0.3*	1	0.5	1.5	52.5
	user43	m	dementia	74	15	30	3	123	NF	0.5*	0.75*	1	0.5	1.5	50
	user02	f	dementia	78	22	24.5	3	84	295	0.4*	0.62*	1	0	1	82.5
	user23	m	dementia	68	20	14.5	3	49	NF	0.5*	0.75*	0	1	1	82.5
	user05	f	dementia	80	25	28.5	3	57	209	0.67*	0.62*	0	0.5	0.5	90
	user21	m	MCI	85	25	29	3	109	NF	0.5*	0.83*	0	0.5	0.5	82.5
	user33	m	MCI	60	26	17.5	1	39	164	1*	1.11*	0	0.5	0.5	95
	user42	f	dementia	79	12	5.5	6	207	NF	0.5*	0.83*	0.5	0	0.5	57.5
	user30	m	dementia	82	21	22	5	137	NF	0.3	0.75	0	0	0	65
	user40	m	MCI	79	28	24	3	109	NF	0.5*	0.75	0	0	0	72.5
regular	Mean	10m /10f	7 MCI / 13 dementia	74.4	23.4	23.0	2.7	87.8	189.3	0.5	0.8	0.7	0.5	1.2	77.8
	Median			77.5	25.0	25.3	3.0	68.0	174.0	0.5	0.8	1.0	0.5	1.5	82.5
	Minimum			58.0	12.0	0.0	1.0	39.0	52.0	0.2	0.3	0.0	0.0	0.0	50.0
	Maximum			85.0	28.0	35.0	6.0	207.0	295.0	1.0	1.7	1.0	1.0	2.0	100.0
all	Mean	20m /20f	12 MCI / 28 dementia	75.0	23.7	23.0	2.6	99.1	184.3	0.5	0.7	0.8	0.7	1.5	78.3
	Median			77.0	25.0	25.5	3.0	72.0	174.0	0.5	0.8	1.0	0.5	1.5	82.5
	Minimum			58.0	12.0	0.0	1.0	31.0	52.0	0.2	0.1	0.0	0.0	0.0	30.0
	Maximum			85.0	28.0	35.0	6.0	329.0	384.0	1.0	1.7	1.0	1.0	2.0	100.0

age [years], **sex** [m=male, f=female], **MCI**=mild cognitive impairment, **MMSE**= Mini Mental Status Examination, **CDT**= Clock drawing Test [Shulman Score], **TMT** = Trail-making Test [sec], **Visual acuity N**= near, **D**= distance, *****corrected with personal glasses,
tasks [0=failure, 0.5=incomplete, 1=completed], **sum sucess**= sum of task A and task B. **NA**= not available, **NF**= not feasible.
