# Supplementary material for: Smartwatch-Based Interventions for People With Dementia: User-Centered Design Approach
Source: JMIR Aging. 2024 Jun 7;7:e50107. doi: 10.2196/50107 (PMC11193079; doi:10.2196/50107)
Supplement: Multimedia Appendix 4 [file aging_v7i1e50107_app4.pdf]

4) Post-Experiment Questionnaire used in intervention study

Questionnaire for use after observational study [SAMi project]

Participants-ID:

Date of trial:

			Strongly agree	Agree	Disagree	Strongly disagree	I can`t tell (neither agree nor disagree)
		coding:	1	2	4	5	3
	**I. Usability**						
scale 1: usability	1.	I could sense the vibration well.	☐	☐	☐	☐	☐
	2.	I could hear the ringtone well.	☐	☐	☐	☐	☐
	3.	The sudden pinging/vibration was disturbing for me. *coding:*	5	4	2	1	3
	4.	I had enough time to notice, that the watch gave me instructions.	☐	☐	☐	☐	☐
	5.	I could recognize prompts (images, text) on the screen well.	☐	☐	☐	☐	☐
	6.	Instructions were shown for an adequate time.	☐	☐	☐	☐	☐
	7.	Based on the readings (images, text) on the watch, I knew what to do.	☐	☐	☐	☐	☐
	8.	I think that I would need the support of a technical person to be able to use the watch.	☐	☐	☐	☐	☐
	9.	I would imagine that I would learn to use the watch very quickly.	☐	☐	☐	☐	☐
	10.	I would like to have more interaction with the watch.	☐	☐	☐	☐	☐
	11.	I could hear the announcements of the watch well.* ** only to be answered by participants in group "intensive"*	☐	☐	☐	☐	☐
	12.	Due to the announcements I knew what I was supposed to do. ** only to be answered by participants in group "intensive"*	☐	☐	☐	☐	☐

			Strongly agree	Agree	Disagree	Strongly disagree	I can`t tell (neither agree nor disagree)
	**II. Design**						
scale 2: form factor	1.	I found the look of the watch appealing.	☐	☐	☐	☐	☐
	2.	I think the size of the watch was proper. ➢ If disagreeing, why not?:	☐	☐	☐	☐	☐
	3.	I found the wristband to be comfortable.	☐	☐	☐	☐	☐
	4.	I was able to mount and dismount the watch independently.	☐	☐	☐	☐	☐
	5.	I found the weight of the watch proper. ➢ if disagreeing, why not?:	☐	☐	☐	☐	☐
	6.	I'd like to wear the watch in my daily life.	☐	☐	☐	☐	☐

Note: items with colored background were inverted retrospectively, when coded (as indicated with numbers in blue circles, p1)

III. Usefulness	Strongly agree	Agree	Disagree	Strongly disagree	I can`t tell (neither agree nor disagree)
scale 3: usefulness					
1. I've got an idea, what benefit such watch could create for me.	☐	☐	☐	☐	☐
2. I think I'd like to use the watch frequently to motivate me for activities.	☐	☐	☐	☐	☐
3. I could imagine that instructions from the watch could create a personal benefit for me.	☐	☐	☐	☐	☐
4. I'd like to have a permanent contact person for service when I would use the watch.	☐	☐	☐	☐	☐
5. I would <u>only</u> use the watch, if my relatives would agree.	☐	☐	☐	☐	☐

IV. Concerns	Strongly agree	Agree	Disagree	Strongly disagree	I can`t tell (neither agree nor disagree)
scale 4: concerns (1)					
1. I found / would find it disturbing that others could hear the announcements from the watch.	☐	☐	☐	☐	☐
2. I found the watch to be too noticeable.	☐	☐	☐	☐	☐
3. I felt comfortable using the watch.	☐	☐	☐	☐	☐
4. Using the watch gave me a feeling of safety.	☐	☐	☐	☐	☐
5. I am worried about data security when using the watch. ➢ if disagreeing, why?	☐	☐	☐	☐	☐
6. I am worried about my health when using the watch. ➢ if agreeing, why?	☐	☐	☐	☐	☐
7. I am concerned that others could think I am needy or dependent.	☐	☐	☐	☐	☐

V. Realization

1. That's the way I want to be noticed from the watch:
(multiple answers accepted)
   - ○ Single vibration
   - ○ Multiple/repeated vibrations
   - ○ Ring tone as tune
   - ○ Ring tone as single tone

2. That's the way I want to be instructed from the watch:
(multiple answers accepted)
   - ○ Speech output
   - ○ Text output
   - ○ Image output
   - ○ Animation

Note: items with colored background were inverted retrospectively, when coded (as indicated with numbers in blue circles, p1)

3. What price would you accept to use the device?
   - ○ up to 50 €
   - ○ up to 100 €
   - ○ up to 200 €
   - ○ up to 500 €

4. Why could you fulfill the instructions from the watch well, or why not?

____
____
____
____
____

5. In your opinion, what need to be changed at the device to be (even more) inviting to use?

____
____
____
____
____

6. In what situations in your daily life would you like to be supported from the watch?

____
____
____
____
____

7. Which additional functions would you like to use at the watch?
   - ○ Emergency button
   - ○ Falls detection
   - ○ Reminder function
   - ○ Navigation / Positioning
   - ○ Clock display
   - ○ Telephone
   - ○ Messages
   - ○ Monitoring of vital parameters
   - ○ Notes
   - ○ Alarm clock
   - ○ Calender
   - ○ Others: ……………………………………………………

VI. Experience	Strongly agree	Agree	Disagree	Strongly disagree	I can`t tell (neither agree nor disagree)
1. I felt stressed during the observation.	☐	☐	☐	☐	☐
2. I forgot that I have been observed during the trial.	☐	☐	☐	☐	☐
3. I would participate again at the study.	☐	☐	☐	☐	☐

Note: items with colored background were inverted retrospectively, when coded (as indicated with numbers in blue circles, p1)

Universität Rostock Traditio et Innovatio

Universitätsmedizin Rostock

Is there something else, you would like to tell us?

Space for notes of the study staff :

Note: items with colored background were inverted retrospectively, when coded (as indicated with numbers in blue circles, p1)
