# Supplementary material for: Smartwatch-Based Interventions for People With Dementia: User-Centered Design Approach
Source: JMIR Aging. 2024 Jun 7;7:e50107. doi: 10.2196/50107 (PMC11193079; doi:10.2196/50107)
Supplement: Multimedia Appendix 5 [file aging_v7i1e50107_app5.pdf]

Supplement

5) Descriptive results of applied questionnaire

The post-intervention questionnaire was administered directly after completion of the interventions. The data set from the post-intervention questionnaire contained very few missing values (~4,4%). After participating in the study, the majority (31 persons) stated that they would be willing to participate again. This demonstrates the participants' dedication to the study. User28 stated: “The procedure was very interesting” and user31 noted that she had been interested in this project as “it is about research”. She and four other participants (user11, user08, user41, user44) ticked “strongly agree” disproportionately often (>mean+1SD). Interestingly, four of those five respondents worked in technical fields during their careers.

Contrary to other empirical studies, we couldn’t see an error of central tendency, despite having an odd number of Likert points. Instead, we observed a strong tendency to the first option: “Strongly agree”, see Figure below.

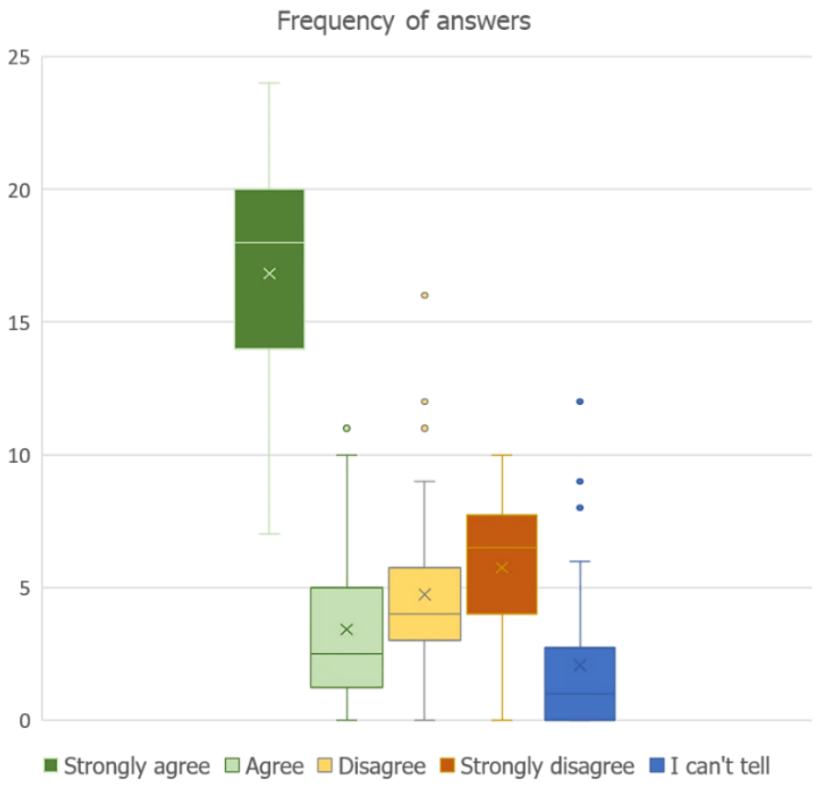

Figure 3s: Frequencies of answers with respect to Likert level from 33 items, n=40 participants.
