# Supplementary material for: Smartwatch-Based Interventions for People With Dementia: User-Centered Design Approach
Source: JMIR Aging. 2024 Jun 7;7:e50107. doi: 10.2196/50107 (PMC11193079; doi:10.2196/50107)
Supplement: Multimedia Appendix 6 [file aging_v7i1e50107_app6.pdf]

Supplement

6) Recommendations for usability questionnaires for PwD

Based on our findings, we may infer the following about the use of questionnaires:

1) use 5-point Likert scales (pro: uneven, not too many choices),
2) use positively and negatively worded items (to control for bias),
3) prefer positively-worded items and start the scale with agreement instead of disagreement,
4) add visual analogs (e.g. emojis or traffic light coding) if applicable,
5) don’t alternate positively and negatively items one after the other,
6) accompany or moderate questionnaires,
7) consider to audio-record questionnaires to capture participants comments.

To assess usability of technology, we suggest to use customized questions focusing on details of your technology, maybe in addition to established scales. Quantitative measures of internal consistency should not be overrated.
